# Step selection techniques uncover the environmental predictors of space use patterns in flocks of Amazonian birds

**DOI:** 10.1002/ece3.1306

**Published:** 2014-11-26

**Authors:** Jonathan R Potts, Karl Mokross, Philip C Stouffer, Mark A Lewis

**Affiliations:** 1 Department of Mathematical and Statistical Sciences, Centre for Mathematical Biology, University of AlbertaEdmonton, Alberta, Canada; 2 Department of Mathematics and Statistics, University of SheffieldSheffield, UK; 3 School of Renewable Natural Resources, Louisiana State University Agricultural CenterBaton Rouge, Louisiana, 70803; 4 Projeto Dinâmica Biólogica de Fragmentos Florestais, INPAAv. André Araújo 2936, Petropólis, Manaus, 69083-000, Brazil; 5 Department of Biological Sciences, University of AlbertaEdmonton, Alberta, Canada

**Keywords:** Amazon rainforest, animal movement, behavioral ecology, home range, insectivorous birds, resource selection, space use, step selection, theoretical ecology

## Abstract

Understanding the behavioral decisions behind animal movement and space use patterns is a key challenge for behavioral ecology. Tools to quantify these patterns from movement and animal–habitat interactions are vital for transforming ecology into a predictive science. This is particularly important in environments undergoing rapid anthropogenic changes, such as the Amazon rainforest, where animals face novel landscapes. Insectivorous bird flocks are key elements of avian biodiversity in the Amazonian ecosystem. Therefore, disentangling and quantifying the drivers behind their movement and space use patterns is of great importance for Amazonian conservation. We use a step selection function (SSF) approach to uncover environmental drivers behind movement choices. This is used to construct a mechanistic model, from which we derive predicted utilization distributions (home ranges) of flocks. We show that movement decisions are significantly influenced by canopy height and topography, but depletion and renewal of resources do not appear to affect movement significantly. We quantify the magnitude of these effects and demonstrate that they are helpful for understanding various heterogeneous aspects of space use. We compare our results to recent analytic derivations of space use, demonstrating that the analytic approximation is only accurate when assuming that there is no persistence in the animals' movement. Our model can be translated into other environments or hypothetical scenarios, such as those given by proposed future anthropogenic actions, to make predictions of spatial patterns in bird flocks. Furthermore, our approach is quite general, so could potentially be used to understand the drivers of movement and spatial patterns for a wide variety of animal communities.

## Introduction

Understanding and quantifying the drivers behind animal movement and space use is a fundamental goal for ecology (Nathan et al. 2008). It is of particular importance in situations where landscapes are changing, making prediction vital for informed conservation (Thomas et al. 2004). The Amazon rainforest is a prime example of a rapidly changing ecosystem, mainly due to wide-scale deforestation (Fearnside 2005; Laurance et al. 2011; Nepstad et al. 2014). The mixed-species insectivore bird communities that live there are key players in the ecosystem, influencing trophic cascades through herbivorous insects and plants (Mäntylä et al. 2011). Therefore, building predictive models of their behavior is of great importance for understanding how to maintain Amazonia's rich biodiversity (Chapin et al. 2000).

These flocks are found in practically all *terra firme* forests in the Amazon basin. They are composed of a wide variety of insectivore species that actively forage in the vegetation (Munn 1984; Powell 1989; Mokross et al. 2014). They spend practically the whole daytime searching the different strata and substrates in the vegetation, with high consumption rates. This makes them important contributors to the species richness of the Neotropical avifauna (Powell 1989).

They typically comprise at least 20 different species at any point in time and may contain as many as 60, with different species making use of various specialist niches found in the forage (Munn and Terborgh 1979). Many species are frequent flock attendants but leave occasionally (flock dropouts), either by switching between flocks, or by having smaller territories than the core species (Jullien and Thiollay 1998). However, each flock has a core composed of 5–10 species that are consistently present and share the same overlapping territory, each breeding pair defending its territory from conspecifics (Munn and Terborgh 1979). In the flocks studied here, the Cinereous Antshrike (*Thamnomanes caesius*) plays a nuclear role by giving alarm and rally calls that maintain flock cohesiveness (Munn 1986). Typically, movement decisions appear to be made by the Cinereous Antshrike, but occasionally, the core species fail to follow and another direction is taken.

Space use for these flocks is very stable with territory shapes changing little in two decades (Martinez and Gomez 2013). The core species gather in the same location at dawn every day, usually in a central position within its territory and will begin foraging from there until sunset where they roost in relatively close vicinity to each other (Powell 1985; Jullien and Thiollay 1998; Martinez and Gomez 2013). The purpose of this paper is to begin a process of disentangling the behavioral drivers behind these movement paths, then to use this understanding to build a predictive model of space use patterns in insectivore bird flocks.

Linking animal movement to space use in a quantitative, analytic fashion is vital for predicting the effects of environmental changes on animal populations (Morales et al., 2010). The factors driving the animals' movement ultimately determine the size and structure of the space that they use in order to meet their everyday needs. By uncovering how these movement processes give rise to spatial patterns, it would be possible to predict the types of terrain that would be used were the environment to be perturbed, by anthropogenic effects or otherwise (Nathan et al. 2008).

In this paper, we make an important step toward this end, by identifying and quantifying some of the key environmental factors that influence Amazonian bird flock movement, then using them to construct a predictive model of space use. Our approach begins by using a step selection function (SSF) (Fortin et al. 2005) to test three hypotheses regarding the drivers behind the flocks' movement decisions. Such techniques, recently reviewed by Thurfjell et al. (2014), have proved invaluable for determining the different drivers of movement in various animal populations. These include foraging decisions in elk (Forester et al. 2009), memory processes in bison (Merkle et al. 2014), mechanisms for coexistence of large carnivores (Vanak et al. 2013), and wolf–ungulate predator–prey interactions (Latombe et al. 2013).

We then derive a master equation from the SSF to link these processes to the emergent space use patterns, following the program started by Moorcroft and Barnett (2008) to integrate resource selection and mechanistic territorial models. The hypotheses we test are that (1) flocks are more likely to move into areas with taller canopies than shorter, (2) flocks tend to move away from higher ground and toward lower, (3) flocks leave some time for the resources to renew before revisiting a tree they have recently visited.

Taller canopies are expected to be preferable for birds as they tend to contain a larger mass of resources (Basset et al. 1992). Furthermore, both leaf abundance and tree height are known to be positively correlated with insect biomass in certain rainforest trees (Ellwood and Foster 2004; Campos et al. 2006). In the system studied here, some birds have been seen moving all the way up to the subcanopy and foraging there (Karl Mokross., Philip Stouffer. pers. obs.), suggesting that the flocks are making use of the entirety of each tree, so benefitting from the greater available biomass in taller trees. On the other hand, lower ground can support more buffered conditions from wind turbulence and sunlight from outside the forest cover (Ewers and Banks-Leite 2013) and naturally hold higher air and soil moisture levels (Baraloto and Couteron 2010) which could potentially increase arthropod loads per vegetation volume (Williams-Linera and Herrera 2003; Chan et al. 2008).

We begin by examining these two covariates in order to develop a basic methodological framework that we can easily extend to build more complicated models. These could include other factors driving the birds' movement decisions, such as memory (Smouse et al. 2010), territoriality (Potts et al. 2013), or tighter movement patterns in dense foliage (Jullien and Thiollay 1998). Building a model one parameter at a time is advantageous as we gain a clear understanding of exactly how, and to what extent, each environmental factor influences flock movement. Although starting with a more complex model may lead to more accurate predictions, it would make it harder to disentangle the relative effects of each model parameter on the resulting space use.

That said, the relative effects of canopy height and topography on movement are interesting in themselves. Indeed, prior to the study, we were unclear whether the two effects were too closely related to be distinguishable. For example, it is known that tree mortality is correlated with steeper slopes in this part of the Amazon (de Castilho et al. 2006), so there is an expectation of more disturbance, hence lower canopies, in steeper areas. Trees may also be shorter on slopes due to leaching of soil minerals. One outcome of the hypothesis testing will be to see whether both parameters are having an individual and separate effect on bird movement, or whether their effects are closely correlated.

## Materials and Methods

### The step selection function model

Our model for bird flock movement is based on a step selection function (SSF) approach (Fortin et al. 2005). Following the formalism initiated by Rhodes et al. (2005), but extended here to take into account correlations in the movement, we write the probability *f* (***x***|***y***, *θ*_0_) of finding an animal at position **x**, having traveled from **y** in the previous step, given that it arrived at **y** on a bearing of *θ*_0_ as follows


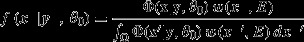
(1)

Here, *w*(***x***, *E*) is a weighting function that depends upon the animal's position **x** and some environmental covariates *E* (Forester et al. 2009), Φ (**x**|**y**, *θ*_0_) is the probability of being at **x** in the absence of habitat selection, given that the animal was previously at **y** and had arrived there on a bearing of *θ*_0_, Ω is the study area, and bearings are measured in an anti-clockwise direction from the right-hand half of the horizontal axis. Each step takes a fixed amount of time *τ*. The function Φ (**x**|**y**, *θ*_0_) allows us to take into account the fact that animals may be more likely to take steps of a particular length, and the distribution of such lengths can be derived from empirical data. For computational purposes, we truncated the step length distribution at steps of >100 m, as these never occur in our data. We include the angle *θ*_0_ into this formulation to allow for the possibility of correlations between successive movement bearings.

For the purpose of testing hypotheses (1) and (2), *w* (***x***, *E*) is a function of the canopy height *C*(***x***) and the topography (i.e., elevation above sea level) *T*(***x***), both measured in meters (m). We test two candidate formulations for *w*(***x***, *E*)


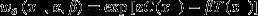
(2)


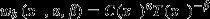
(3)

Notice that eq. 3 can also be written as 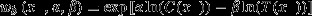, in keeping with the original formulation of the step selection function from Fortin et al. (2005). As we would expect the birds to be more likely to move toward lower ground than higher, we place a minus sign before the *β* in each equation, so that *β* is expected to be positive. We treat canopy height and topography as two separate variables, noting that there is little or no correlation between the two (*R*^2^ = 0.007).

To test hypothesis (3), we assume that the resource amount (i.e., insect biomass) at the start of the day (*t* = 0) is proportional to the canopy height. This relationship was observed by Campos et al. (2006), who gave linear relationships between tree height and biomass for various insect species. As the birds move through an area, they deplete the resources, which take a time *Gτ* to renew. The resource amount present at a site at time *gτ* after having been visited is assumed to be *R*(***x***, *t*, *G*) = *gC*(***x***)/*G* as long as *g* < *G*, otherwise *R*(***x***, *t*, *G*) = *C*(***x***). Here, *t* is the time since start-of-day, and a unit of resources is implicitly defined to be the maximum amount of usable resources sustainable by a tree per meter of tree height. At time *t* = 0, we assume *R*(***x***, 0, *G*) = *C*(***x***). As with hypotheses (1) and (2), we test two candidate formulations for *w*(***x***, *E*)


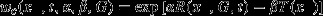
(4)


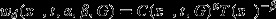
(5)

Notice that when *G* = 1, we have 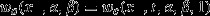 and 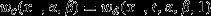.

### Data collection methods

Flock activity is conspicuous, allowing birds to be followed on foot. As flocks moved, geolocations were recorded at 30-sec intervals with a hand-held GPS unit (Garmin Vista HCX, equipped with Wide Area Augmentation System coverage ensure reliable precision under canopies). The observer maintained a distance of 10–20 m from the flocks to ensure no alarm or avoidance behavior was induced in the birds. Observer distance is not in perfect lockstep with the flock, yet the average distance to the approximate center of the flock could be maintained to an accuracy of a few meters. Where possible, we used the location of a Cinereous Antshrike as the flock location. This species was usually conspicuous in the center of the flock. Other more active species typically spread out over a radius of 5–10 m, depending on the size and speed of the flock.

Although GPS error can be around 10 m, it is mainly caused by the relatively slow movement of the ionosphere (Parkinson and Spilker 1996) which only changes by a few centimeters during 30-sec intervals. Indeed, evidence from using hand-held GPS for tracking butterflies suggests that the median drift (i.e., absolute error) between consecutive 15-sec relocations is only 8 cm (Severns and Breed 2014). Therefore, it is reasonable to assume that the measured step lengths and turning angles accurately reflect reality.

Compared to other available methods, these data reflect well the movement of flocks on a small spatio-temporal scale. They provide a high resolution of time sequence that is not possible in radio-telemetry studies, and presently, no other techniques allow the gathering of detailed spatial data for passerines of this size. Unlike remote telemetry, this method also allows the direct observation of behavior, so the observer can directly verify whether the recorded spatial locations are corresponding well with the canopy height and topographical maps.

For measuring canopy heights, we used a LIDAR (Light Detection and Ranging) canopy height model (CHM). Similarly, topography (Digital Elevation Models DEM) was acquired using small footprint airborne LIDAR. The derived (postprocessed) images from the LIDAR data are 1 m/pixel resolution, which we transformed into 10 m lattices by bilinear interpolation. LIDAR data were collected by airborne laser scanning using a Hexagon-LEICA ALS50 PHASE II MPiA sensor of 150 kHz, at 800 m altitude, with 24 degrees opening, 118 MHz pulse rate, 58 Hz scan rate, 3,7 points/m^2^ density. Swaths were of 340 m wide, spaced at 240 m. Postprocessing used a forest service methodology to generate DEM and CHM at 1 m^2^/pixel [see Stark et al. (2012) for more details on LIDAR data collection and analysis].

Sampling was restricted to areas within LIDAR coverage which did not span more than 1.5 km^2^, and of which five were scattered along the study area at an average of 6 km from each other. The choice of flocks was mainly restricted to locations where the entire home range would be inside this LIDAR coverage (i.e., away from edges of the canopy height models and digital elevation models). We analyzed six different flocks from the Dimona LIDAR dataset, which was the largest (2.8 km by 1.5 km) and best-sampled area, and also the one that presented the highest variability in vegetation types. This area falls within the Biological Dynamics of Forest Fragments Project (BDFFP), about 70 km north of Manaus, Brazil (see http://pdbff.inpa.gov.br/ for maps).

Data were gathered during the dry seasons between June and November during 2009–2011, and each flock was tracked for between 5 and 11 days. Each flock gathers in one particular place each day, then moves around the forest for a total of about 11.5 h during the day, before each bird goes back to its roost for the night. Flock composition was sampled every half hour to check that cohesiveness was being maintained. Flocks were taken from a variety of different habitat types to ensure the greatest generality in our findings and minimize the effects of spatial autocorrelation. These included areas predominated by secondary forests, areas of primary forest away from edges, and areas near forest edges. Flocks were initially found based on their dawn gatherings. As they were first followed, it was unclear where they would go, so it is unlikely there was a bias to the flocks chosen. If the flocks moved into areas that were difficult for the observer to reach, bearings in relation to the observer were taken in order to make the necessary corrections in the data. In these cases, the observer did not lose the flock from sight.

### Parametrizing the models from the data

The first step in parametrizing the models is to calculate the step length and turning angle distributions, that is, the distance between successive positions and the angle an animal turns through from one move to another, respectively [see e.g. Crist et al. (1992)]. As these depend upon the temporal resolution *τ* (i.e., the time between successive position fixes), we use both *τ* = 1 min and *τ* = 5 min, deriving two different sets of step length and turning angle distribution for the different values of *τ*. The value *τ* = 1 min is chosen because bird flocks tend to move from one tree to another at an average of approximately every 1 or 2 min. Although their movement is a continuous rather than discrete process, the model is formulated so that this timescale roughly represents the small-scale decisions that the birds make regarding whether they stay in a tree or choose to move to another. We also examine the case *τ* = 5 min to determine whether the decisions about where to move can instead be viewed as taking place on a timescale longer than a single jump between trees. In other words, the birds might only be considering the next tree they move to when deciding where to go (*τ* = 1 min), or they might be thinking a few trees ahead when they make this decision (*τ* = 5 min).

The step length distributions are fitted to both a Weibull distribution (Forester et al. 2009) and an Exponentiated Weibull (EW) distribution (Nassar and Eissa 2003), using the Akaike information criterion (AIC) to determine the best model, whereas we fit the turning angles to a von Mises distribution (Marsh and Jones 1988). The Weibull, EW, and von Mises distributions have the following forms, respectively:


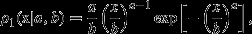
(6)


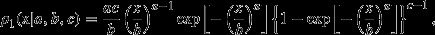
(7)


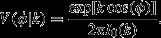
(8)

As the rainforest canopy consists of distinct treetops whose widths are each roughly 10 *m* across, we split the terrain Ω into a grid *S* of 10 *m* by 10 *m* squares. This allows us to associate a value of *C*(*s*) and *T*(*s*) to each square *s* in *S*, respectively, the mean canopy height and mean topography of the square. Canopy heights ranged from 50 m to essentially none, which we set to be 1 m for the purpose of the model (a value of zero meters for the canopy height would give an identically zero probability of moving there, which is biologically implausible). Topography ranged from 40 to 115 m. Parametrizing eq. 1 from the data therefore requires maximizing the following likelihood function


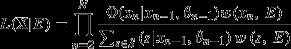
(9)

where **X** = {***x***_0_, ***x***_1_, …, ***x***_*N*_} are the consecutive positions of a flock, *θ*_*n*_ is the bearing from ***x***_*n*−1_ to ***x***_*n*_, ϕ is the product of the best-fit step length and turning angle distributions, and *w* is either *w*_*a*_, *w*_*b*_, *w*_*c*_, or *w*_*d*_, depending on which model we are fitting.

To test hypothesis (1), we fix *β* = 0 and find the value of *α* that maximizes L(**X**|*E*), which we call *α*_*m*_. We then use the likelihood ratio test to compare the resulting value of L(**X**|*E*) with the value of L(**X**|*E*) when both *α* and *β* set to zero. For hypothesis (2), we fix *α* = *α*_*m*_ and find the value of *β* that maximizes L(**X**|*E*), again using the likelihood ratio test to compare this value of L(**X**|*E*) with the one where *α* = *α*_*m*_ and *β* = 0.

This technique of fixing *α* = *α*_*m*_ when testing hypothesis (2) means that we are only testing for topographical effects on movement that are *additional* to the effects of canopy height. This is to address the question of whether these effects can be separated (see the last paragraph of the Introduction). We then find the values of *α* and *β* that maximize L(**X**|*E*) by varying both parameters simultaneously, giving best-fit values denoted by *α*_bf_ and *β*_bf_. We use a Markov bootstrap method with 100 bootstraps to find standard errors for *α* and *β* (Horowitz 2003).

Hypothesis (3) is tested by fixing *α* = *α*_bf_ and *β* = *β*_bf_ and finding the value of *G* that maximizes L(**X**|*E*), then using the likelihood ratio test to compare the resulting value of L(**X**|*E*) with the value of L(**X**|*E*) when *G* = 1. For each maximization calculation, we use the Nelder-Mead simplex algorithm (Lagarias et al. 1998), as implemented in the Python maximize() function from the SciPy library (Jones et al. 2001).

### Constructing the space use distribution

We use two methods for constructing the space use distribution from the parametrized SSF (eq. 1), via simulation analysis and through constructing the master equation and numerically deriving its steady-state solution. For the former approach, we simulate one particular flock's movement on the grid *S* using the jump probabilities given by SSF. As the flock gathers in one particular place each day and moves around the terrain for a total of about eleven-and-a-half hours during the day, we start the simulated birds at the gathering point and run the simulation for 138 time steps, each step representing *τ* = 5 min (giving 11 h 30 min in total), taking a note of all the positions at which the flock landed after each step. We repeat this 100 times, representing 100 days, giving 13,800 simulated positions in total. In the data, we tend to have around 10 days per flock. However, we use 100 here to average out some of the stochasticity. From these simulated positions, we calculate the 50%, 60%, 70%, 80%, and 90% Kernel density estimators (KDEs), using a fixed kernel method with smoothing parameter *h* = *σn*^−1/6^ where 
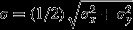
 and *σ*_*x*_, *σ*_*y*_ are the standard deviations of the simulated data in the *x*- and *y*-directions, respectively (Worton 1989). KDE calculations are performed using Python.

In addition to simulation analysis, we also construct the master equation for the probability density function *u* (***x***, *θ*, *t*) of the animal being at **x** at time *t* having traveled there on a bearing of *θ*. This allows us to compare our results with the predictions of Barnett and Moorcroft (2008), who mathematically analyzed the step selection function (eq. 1) in the simpler case where the turning angle distribution is uniform. They proved that the steady-state (time-independent) solution u_*_(***x***) is proportional to 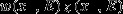, where 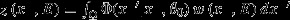 is a local averaging of *w* (***x***, *E*). We examine to what extent this result extends to our more complicated situation of a correlated random walker. We use eq. 1 to construct the following master equation


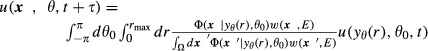
(10)

where *y*_θ_(*r*) describes the locus of points **y** upon which the animal could approach **x** = (*x*_1_,*x*_2_) at bearing θ, that is, *y*_*θ*_ (*r*) = (*x*_1_ + cos (*θ* + *π*)*r*, *x*_2_ + sin (*θ* + *π*)*r*), with *r* denoting the distance between *y*_*θ*_ (*r*) and **x** (Potts et al. 2014). Here, *r*_max_ is the distance along this line from *x* to the boundary of Ω and so gives the upper endpoint of integration. To calculate the steady-state distribution, we solve eq. 10 iteratively until |*u*(*x*, *θ*, *t* + *τ*) − *u*(*x*, *θ*, *t*)| < 10^−8^ for every value of **x** and *θ*. The area Ω for this calculation is defined to be the 95% KDE of the flock used for the simulations. We used zero-flux boundary conditions, which models the fact that the birds are confined within their territory. Calculations were coded in C and it took approximately 2 h to find a single steady-state distribution.

Note that in these methods, we are separating the fitting of the turning angle and step length distributions from the fitting of the weighting functions. This makes the maximization procedure far faster and means the algorithms are more likely to converge to the global maximum. However, if the weighting function *w* gives a particularly strong selection for an environmental covariate and/or the step length distributions are fat-tailed, then this separation may cause inaccuracies in the resulting model. To test that this is not the case, we calculated the mean and standard deviation of the step length and turning angle distributions from the above simulations to verify that the weighting function had not significantly altered them.

## Results

### Step length and turning angle distributions

**Figure 1:**
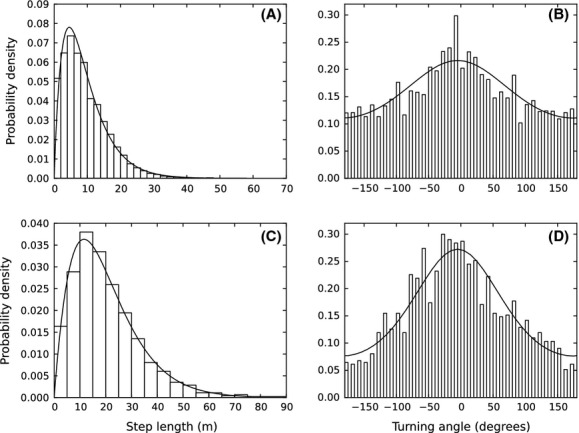
Step length and turning angle distributions. Panel (A) shows the empirical step length distribution (bars) for data where the temporal resolution is *τ* = 1 min, together with the best-fit Exponentiated Weibull distribution (solid curve). The latter is given in eq. 7, with *a* = 1.06, *b* = 6.90 and *c* = 1.82. The bars in panel (B) denote the empirical turning angle distribution for the same data, whereas the curve denotes the best-fit von Mises distribution, given in eq. 8 with *k* = 0.336. Panels (C) and (D) are analogous to (A) and (B), respectively, except they use the dataset where *τ* = 5 min, rather than *τ* = 1 min. Here, *a* = 1.26, *b* = 17.2, *c* = 1.55, and *k* = 0.637.

For both cases *τ* = 1 min and *τ* = 5 min, the best-fit step length distribution is an Exponentiated Weibull (EW) distribution (Fig. 1). For *τ* = 1 min, ΔAIC = 126.9 between EW and Weibull. For *τ* = 5 min, ΔAIC = 14.6. The step length distributions both increase from 0 *m* initially, before decaying (Fig. 1). However, this is not an indicator that birds are more likely to move a medium length distance than a very short distance, but is simply due to there being less area in the annulus of radius between *r* and *r* + *δr* when *r* is smaller. If *δr* is small, then the total amount of area into which a flock can move, given that it moves a distance between *r* and *r* + *δr*, is approximately *δr* × 2*πr*, which is proportional to *r*. To find the relative preferences of the birds to move a particular distance, it is therefore necessary to divide the probability density, *P*(*r*), by the distance moved, *r*. If we do this for our data on the 1 min temporal resolution, we find that *P*(*r*)/*r* is approximately 0.044 exp(−*r*/4.75) and for the 5 min timescale *P*(*r*)/*r* ≈ 0.0080 exp(−*r*/11.3), both of which decay monotonically as *r* increases.

### Hypothesis testing

The tests indicate that there is a significant effect of both canopy height (hypothesis 1) and topography (hypothesis 2) on the flocks' movement (Table 1). Furthermore, these aspects of the landscape each affect bird movement separately, rather than being highly intertwined. However, accounting for resource renewal, so that birds are less likely to revisit trees that they have recently visited, does not improve the model fit (hypothesis 3). The conclusions are the same both for *τ* = 1 min and *τ* = 5 min, so we cannot conclude anything about the temporal resolution on which decisions are made.

**Table 1:** Results of hypothesis testing. The first column is number of the test, as given in the introduction. This test finds the best-fit parameter given in the second column. The third column denotes the weighting function used for the test (see Eqs. 2–5) and the fourth gives the value of the time *τ* between successive position measurements in the data. The fifth column shows the value of the parameter that fits the data best (±standard error), with a *P*-value from the likelihood ratio test (see Methods) given in the sixth column and the results of a 1% significance test in the final column (note that a 5% test would give identical results)

Test	Parameter	*w*-function	*τ* (mins)	Best fit	*P*-value
1	*α*	*w*_*b*_	1	0.095 ± 0.037	0.0038
2	*β*	*w*_*b*_	1	1.658 ± 0.345	<0.001
3	*G*	*w*_*d*_	1	1.00	N/A
1	*α*	*w*_*b*_	5	0.227 ± 0.065	<0.001
2	*β*	*w*_*b*_	5	1.697 ± 0.436	<0.001
3	*G*	*w*_*d*_	5	1.00	N/A

To put these in a biological context, consider two trees, equally accessible over a 5-min interval and on ground of equal elevation, but one *A*% taller than the other. For example, if one is 30 m high and the other 20 m high then *A* = 50. Then, the birds are (1 + *A*/100)^0.277^ = 1.5^0.277^≈1.1 times more likely to move to the taller tree than the shorter, that is, about 10% more likely. The effect is more dramatic when considering the difference between a completely deforested area with essentially no canopy (which we set to 1 m for the purposes of the model) and primary forest with, say, 30 m canopy. Here, *A* = 3000, so the flocks will be around 160% more likely to move to the primary forest.

Conversely, suppose that both trees are of equal height but one tree is ground *B*% higher above sea level than the other. Then, the birds are (1 + *B*/100)^1.697^ times more likely to move to the tree on lower ground. For example, an decrease from 50 to 40 m elevation leads to a 1.25^1.697^≈1.460 increase in probability of moving there, that is, they are 46% more likely to move to the 40 m elevation.

The weighting function *w*_*b*_ (eq. 3) provides a better fit to the data than *w*_*a*_ (eq. 2) for *τ* = 5 min. The AIC for *w*_*b*_ is lower than that for *w*_*a*_ (ΔAIC = 3.8). Although the AIC for *w*_*b*_ for *τ* = 1 min is slightly lower than for *w*_*a*_ (ΔAIC = 0.1), the change in AIC is not large enough to be considered good evidence that *w*_*b*_ is better than *w*_*a*_. In Table 1, we detail the results for the function *w*_*b*_ and its generalization *w*_*d*_ (eq. 5). Results for *w*_*a*_ and *w*_*c*_ (eq. 4) are qualitatively similar.

### Space use distributions

**Figure 2:**
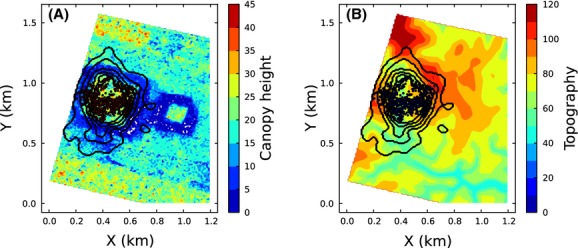
Plots of simulated and real data. Both panels show the empirical data for one flock (dots) together with the 50%, 60%, 70%, 80%, and 90% kernel density estimation curves for the simulated data (black curves). See the Methods section for details on how the simulations were performed. The colors underlying panel (A) denote the canopy height, whereas in panel (B) they give the topography, that is, height of the ground above sea level.

Figure 2 compares the simulated space use with the empirical data on flock positions. The KDE contour lines for the simulated data are quite tightly packed around the edge of the empirical data points, suggesting that the model is giving a reasonable prediction of space use patterns. However, the extent of the simulated home range is clearly larger than the empirical home range.

Although separating the fitting of the step length and turning angle distributions from the environmental interactions may mean that the fit is less accurate than if all parameters were fitted together, it turns out that the mean of the simulated data's step length distribution is 20.05 ± 0.95 m (95% confidence intervals), compared with 20.09 m from the data. The standard deviation of the simulated step lengths is 13.55 ± 2.01 m as compared with 13.23 m from the data. Similarly, the standard deviation of the turning angles from simulation output is 82.1 ± 8.7 degrees as compared with 82.7 degrees from the data, and the mean is −0.2 ± 6.9 degrees, as compared with −1.7 degrees from the data. Therefore, including the weighting function does not significantly change the step length or turning angle distributions.

### Comparison with analytic results

**Figure 3:**
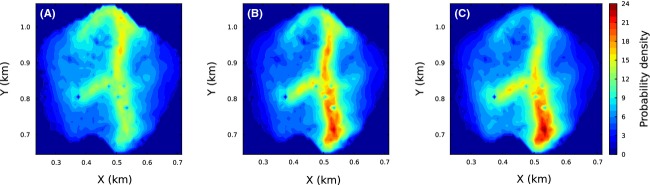
Exact and approximate steady-state solutions of the master equation. Panel (A) shows the numerical steady-state solution of our master equation (eq. 10) with *w* = *w*_*b*_ (eq. 3) and the parameters that best fit the data (see Fig. 1 and Table 1). The numbers on the axes correspond to those in Figure 2 for ease of comparison. The analytic solution, given in Barnett and Moorcroft (2008, eq. 13), is given in panel (B). Although there are some similarities between panels (A) and (B), the approximation is evidently not particularly good. However, when we replace the von Mises turning angle distribution with a uniform distribution, the numerical steady-state solution of eq. 10 (panel C) is visually very close to that of panel (B), as expected.

Previous work showed that if there is no correlation in an animal's movement, the steady-state space use distribution is proportional to *w*(***x***, *E*)*z*(***x***, *E*) as long as the turning angle distribution is uniform (Barnett and Moorcroft 2008; eq. 13), where *z*(***x***, *E*) = ∫_*Ω*_ Φ(***x***′|***x***, *θ*_0_)*w*(***x***, *E*)*dx*′. By numerically deriving the steady space use distribution for our model, we show that this result breaks down when we include correlation in the movement process. Figure 3A and B compare the analytic result to the numerical one in the specific example of our Amazonian bird flock model, in the case 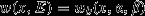 (see eq. 3). However, if we assume that the turning angle distribution is uniform, then the analytic solution is very similar (Fig. 3B and C).

## Discussion

We have constructed a step selection function (SSF) to test three hypotheses about the drivers behind Amazonian bird flock movement decisions. We found that flocks have a tendency to move toward areas covered by higher canopies, but move away from areas of higher ground. The preference for higher canopies is likely to be due to the greater abundance of resources, through enhanced microclimatic conditions in the understory and more foraging substrate (Basset et al. 1992). Lower ground is likely to be preferred because it has a moister environment that can hold a higher insect biomass (Chan et al. 2008; Bueno et al. 2012). Although these aspects are related, we found no evidence of correlation between topography and canopy height, and each appears to have their own separate effects on flock movement.

The flocks appear to be just as likely to move back to a place that they have recently visited than one that they have not visited for a while. This suggests that when they visit a tree, they do not deplete the resources completely, but leave the tree in the knowledge that there is still food to be found there. While it may seem advantageous to stay at a tree as long as it is profitable to do so, in order to conserve energy (Houston et al. 1993), this frequent movement from tree to tree might be a tactic to avoid predators. Alternatively, insects may temporarily be adopting cryptic behavior on the presence of birds, thus forcing the birds to move on quickly as insects become rapidly harder to find.

We tested different functional forms for the selection weighting, something that is rarely done in literature on step selection functions but could be important (Lele et al. 2013). Although we would be surprized if the functional form were to change the outcome of hypothesis testing, it could very much affect the resulting parameters that are used to build the mechanistic model. For example, an exponential effect of the canopy height vastly increases the relative attraction to very high canopies as compared with a power law effect, as this is effectively the difference between a linear and a logarithmic scaling (see the note after eq. 3). This has the potential to vastly change the predicted space use patterns. Therefore, it is vital to consider functional form when using step selection techniques to build mechanistic models.

Our SSF approach enabled us to run simulations that were used to predict the utilization distribution (UD) of a flock, thereby relating the small-scale movement decisions to the large-scale space use patterns. While the resulting simulated UD captured certain qualitative aspects of the empirical data (Fig. 2), it overestimated the home range size. In comparison, a straightforward random walk model, based on the empirical mean step length distribution, would give a normal distribution with the 90% contour approximately 395 m from the gathering point. This contour would overlap the corresponding (outer) contour from Fig. 2, but would be circular, whereas the simulation contour is far from symmetric. Therefore, although certain features of space use are being predicted by our model, there must be some other aspect of the birds' movement decisions keeping them far more spatially confined than our current model predicts.

We propose two plausible mechanisms that might explain this confinement. First, these flocks are highly territorial (Develey and Stouffer 2001), so interactions with neighboring flocks may cause each flock to use less space than they would otherwise. The mechanism of conspecific avoidance has been shown to give rise to spatial confinement in various species of canid (Lewis and Murray 1993; Moorcroft et al. 2006; Potts et al. 2013). These all deal with avoidance via scent marking, whereas territories in birds are defended via vocalizations and direct interactions (Munn and Terborgh 1979). However, the generic modeling framework from Potts et al. (2013) could be used to construct coupled SSFs, whose weighting functions *w* depend both upon the position of the individual and on interactions with neighbors. These interactions may either be direct or mediated by vocal, visual, or olfactory cues.

Second, memory effects, with birds having a preference to move back toward places they have frequently visited, can cause spatial confinement. Theoretical studies by Briscoe et al. (2002) have described such a mechanism in wolf (*Canis lupus*) populations, and the general results of Tan et al. (2001) show that memory can severely constrain the amount of area used in a given time period. Although it is tricky to determine empirically what constitutes a bird's cognitive map of the environment, it is generally considered that memory is an important factor in the spatial confinement and site-fidelity of many animals (Smouse et al. 2010).

By turning our SSF into a master equation for the spatiotemporal probability distribution of the flock's position, we compared our results to a recent approximate analytic prediction by Barnett and Moorcroft (2008) that applies when the turning angle distribution is uniform. However, their results fail whenever there is correlation in the animal's movement at any timescale, a fact noted by Barnett and Moorcroft (2008) but not emphasized in their ecologically motivated paper Moorcroft and Barnett (2008). The more the correlation, the worse the prediction is likely to be, so it is necessary to take care when applying these results to empirical data. Although the correlation in the birds' movement greatly affected the movement patterns, when we removed any intrinsic correlation from our movement model, the predictions of Barnett and Moorcroft (2008) were visually very good (Fig. 3).

Although our results are not testing conservation decisions *per se*, the application of these models could provide basis for informed management decisions for a subset of the avian community that is known to be very sensitive to forest disturbances. By providing information on how a combination of two important habitat features influences habitat use and how these flocks anchor their home ranges, this would allow for more realistic estimations of areas that are more important to these species. Also, the drivers related to resource abundance and renewal provide important insights into the nature of the relationship of insectivorous birds and their resource, a topic that has challenged researchers for years (Sherry 1984; Şekercio lu et al. 2002). These results also have the potential to be extended to closely related species in other regions of Amazonia. For example, in southwestern Amazonia, flocks are lead by *T. schistogynus* rather than *T. caesius* (Munn and Terborgh 1979) which may behave differently. It is an interesting future challenge to analyze these differences rigorously. The dynamic and collective nature of bird flock decisions is also likely to have an impact on behavioral decisions. As we refine our model to make it more accurate at predicting space use, it will likely be necessary to take these effects into account.

## Data Accessibility

Data can be found in Dryad doi:10.5061/dryad.47jh1.
